# Acute complicated jejunum diverticulitis: a case report with a short literature review

**DOI:** 10.3389/fmed.2024.1413254

**Published:** 2024-05-15

**Authors:** Stefan Chiorescu, Mihaela Mocan, Maria Elena Santa, Florin Mihăileanu, Roxana Mihaela Chiorescu

**Affiliations:** ^1^Department of Surgery, Iuliu Hațieganu University of Medicine and Pharmacy, Cluj-Napoca, Romania; ^2^Department of Surgery, Emergency Clinical County Hospital, Cluj-Napoca, Romania; ^3^Internal Medicine Department, Iuliu Hatieganu University of Medicine and Pharmacy, Cluj-Napoca, Romania; ^4^Department of Internal Medicine, Emergency Clinical County Hospital, Cluj-Napoca, Romania; ^5^Department of Hematology, Oncology Institute "Prof. Dr. Ion Chiricuta ", Cluj-Napoca, Romania

**Keywords:** intestinal perforation, jejunal diverticulosis, diverticulitis, segmental resection, surgical treatment

## Abstract

**Introduction:**

Jejunal diverticulosis is a rare condition. Most of the time, it is asymptomatic; but it can cause severe complications such as intestinal perforation, mechanical occlusion, and hemorrhage.

**Case presentation:**

A patient aged 78 years, with a history of biological aortic valve prosthesis, atrial fibrillation, type 2 diabetes mellitus, and chronic obstructive pulmonary disease, presented in the emergency department for acute abdominal pain in the lower abdominal floor, nausea, and inappetence. Abdominal computed tomography revealed an inflammatory block in the hypogastrium, agglutinated small intestinal loops, fecal stasis, and air inclusions. Pulled mesentery and associated internal hernia are suspected. Exploratory laparotomy was performed, revealing an inflammatory block in the hypogastrium, whose dissection revealed inner purulent collection and the appearance of jejunal diverticulitis, a diagnosis confirmed by histopathological examination. Segmental resection of the jejunum with double-layer terminal–terminal enteroenteric anastomosis, lavage, and drainage was performed. The evolution was favorable.

**Conclusion:**

Based on our brief review, the diagnosis of complicated jejunal diverticulosis is difficult and sometimes not accurately established, even by high-resolution imaging techniques, with diagnostic laparotomy being necessary for these situations. Surgical treatment should be considered before severe complications develop.

## 1 Introduction

Jejunal diverticulosis is a rare pathology that occurs in 0.3–1.3% of patients (1). Most often, it is asymptomatic; but sometimes it can give serious complications such as diverticulitis, perforation, mechanical occlusion, and hemorrhage. Due to its position, most of the time, high-evolution imaging techniques cannot establish the diagnosis. Therefore, this diagnosis should be considered in patients with intense abdominal pain localized periumbilically or in the hypogastrium. Performing an emergency laparoscopy is preferable to a conservative medical attitude in these situations. We present the case of a 78-year-old woman who presented with a complication of small intestine diverticulosis – diverticulitis and intestinal perforation requiring emergency surgery.

This paper aims to describe a case of small intestine diverticulitis and intestinal perforation and review the cases of small intestine diverticulitis published in the last 10 years to determine the best method of diagnosis and appropriate conservative or surgical treatment for these cases.

## 2 Case presentation

A 78-year-old female patient was referred to our emergency department with complaints of lower abdominal pain, slowed gastrointestinal transit, nausea, loss of appetite, and fatigue.

Her medical history was significant for hypertension, biological aortic valve, mitral valve regurgitation, atrial fibrillation, heart failure NYHA II/III, chronic obstructive pulmonary disease Gold II, type 2 diabetes mellitus, and dyslipidemia. There was no relevant family history. Her medication history consisted of Perindopril 10 mg/Indapamide 2.5 mg, Acenocoumarol 2 mg/day, Digoxin 0.25 mg/day, 5/7 days, Budesonide 160 microg/Formoterol 4.5 micron 2× two puffs/day, and Metformin 1,000 mg/day.

The pain was moderate without irradiation. There were no aggravating or relieving factors. The symptoms were worsening in the last 4 days before admission.

On admission, the patient had a body temperature of 37.7°C, a pulse rate of 80 beats/min, a blood pressure of 140/80 mmHg, and a saturation of 96% in ambient air. Physical examination revealed a new periumbilical mass associated with lower abdominal tenderness but no rigidity or rebound tenderness.

Blood tests showed leukocytosis (22 × 109/L), neutrophilia (20.09 × 109/L), C-reactive protein >30 mg/dL, procalcitonin 9.5 ng/mL, creatinine = 3.42 mg/dL, urea = 127 mg/dL, and INR > 9.

**Figure 1:**
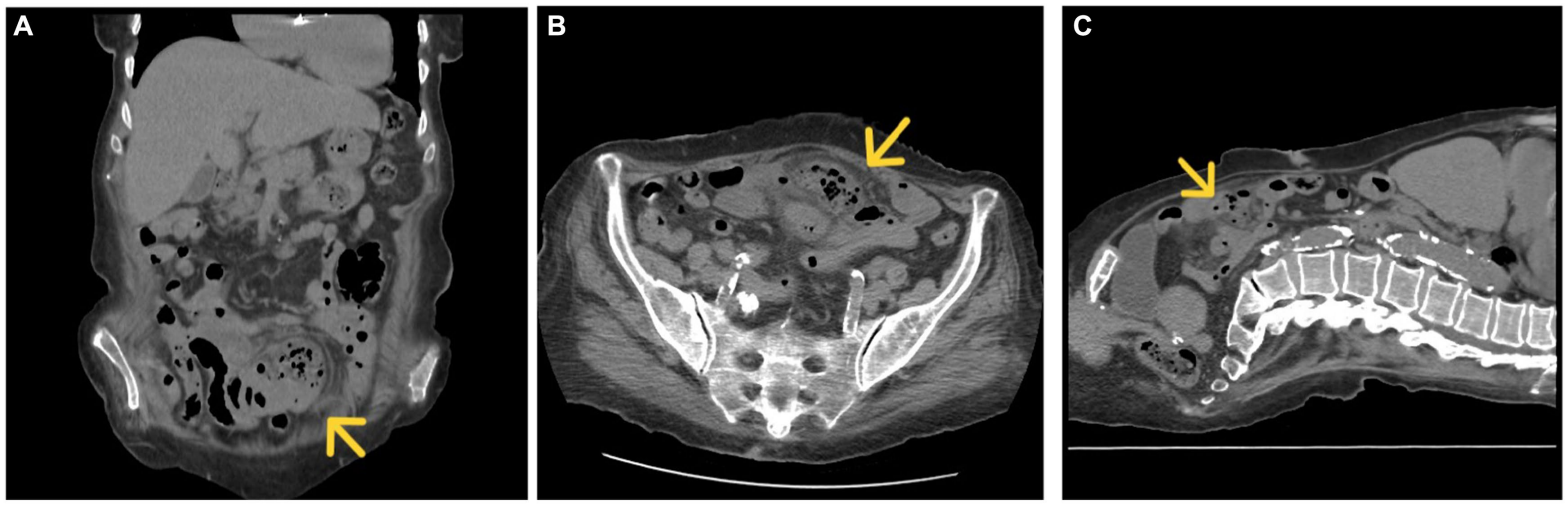
CT, coronal **(A)**, axial **(B)**, and sagittal section **(C)** inflammatory block marked with a yellow arrow.

An abdominal ultrasound was performed, which revealed intestinal loops with peristalsis present at the level of the descending colon – a slightly dilated intestinal loop with a slightly thickened intestinal wall. We completed with abdominal and pelvic computer tomography scan (CT), which showed an inflammatory block at the level of the hypogastrium – thin intestinal loops, agglutinated, forming a lesional block of 90/69/60 mm, with fecal stasis and air inclusions. Adjacent fat infiltrated, with multiple fluid fuses present. Pulled mesentery and mesenteric vessels – an associated internal hernia is suspected (Figure 1).

An echocardiogram reported neurofunctional biological aortic valve, concentric left ventricular hypertrophy, and mitral valve regurgitation. There were no vegetations of valves observed.

**Figure 2:**
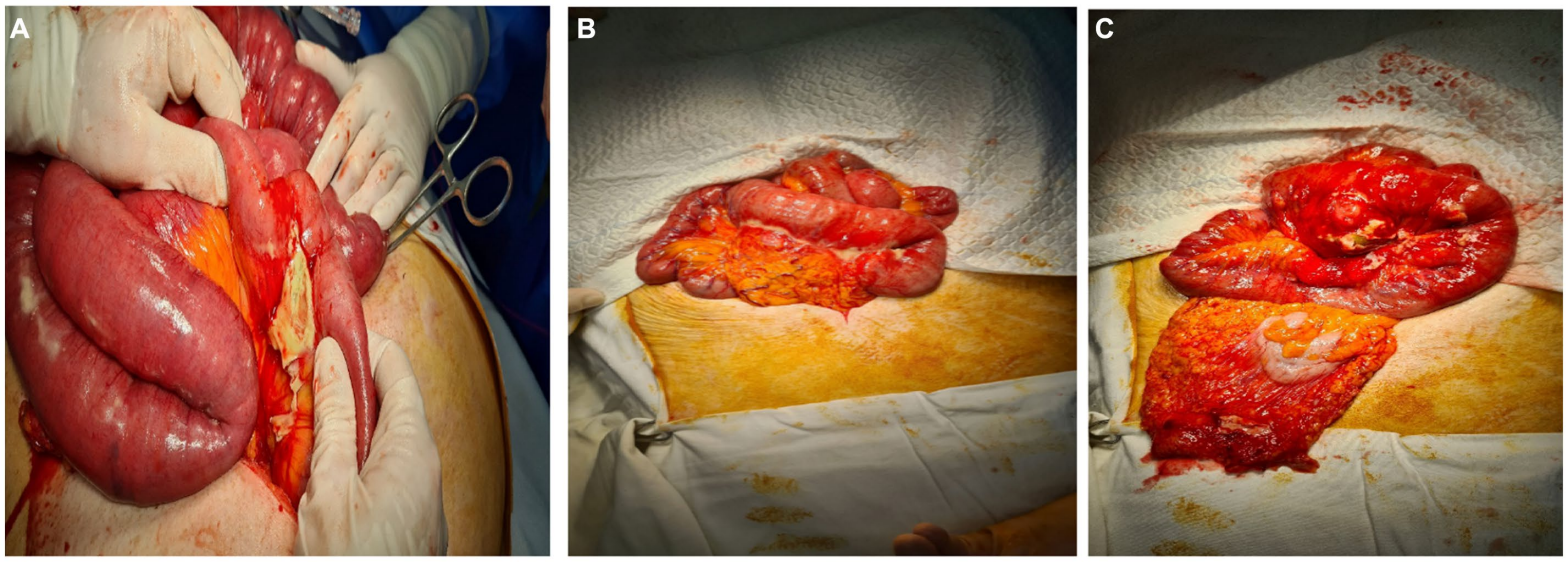
**(A–C)** Intraoperative appearance. Intestinal inflammatory block, the dissection of which reveals an abscessed jejunal diverticulum.

Due to high suspicion of intestinal subocclusion, the patient underwent exploratory laparotomy via a median incision. The abdominal cavity was explored, detecting an epigastric inflammatory block that includes several loops of the small intestine (jejunum and ileum) and omentum, the dissection of which reveals the minimal interileal purulent collection and a tumor at about 50 cm from the duodenojejunal angle, with the appearance of diverticulitis. At 40 cm from the duodenojejunal angle, another uncomplicated intestinal diverticulum of about 2 cm diameter is identified (Figure 2).

We performed segmental resection of the jejunum with terminal–terminal enteroenteric anastomosis.

in a double layer, lavage, and drainage. IV fluids, Ceftriaxone 2 g/day once daily, Metronidazole 500 mg every 8 h, probiotics, and Famotidine 20 mg were administered. The treatment of associated diseases continued.

On Day 4, the patient had normal gastrointestinal transit. During hospitalization, the patient presented one episode of upper gastrointestinal bleeding. The patient underwent an esogastroduodenal endoscopy, which revealed a Forrest III antral gastric ulcer and congestive corporeal gastritis without an active source of bleeding. Under treatment with proton pump inhibitors, erythrocyte mass transfusion evolution was favorable. The patient was discharged home on day 21.

Histopathological examination confirmed the diagnosis of jejunal diverticulitis. Stasis, hemorrhagic areas, and lymphoid follicles were observed at the resection margins. The intestinal wall shows areas of necrosis spread throughout its thickness, hemorrhagic areas, abscesses, and a marked transparietal predominantly neutrophilic inflammatory infiltrate. At the level of the diverticulum, fibrin–hematic exudate is observed at the level of the serosa, vascular stasis, hemorrhagic areas, and mixed inflammatory infiltrate, with the presence of lymphoid follicles and erosions at the level of the mucosa (Figures 1, 2).

## 3 Discussion

Diverticula are hernias of the mucosa and submucosa through the muscular layer of the intestinal wall. It is usually located in the sigmoid and descending colon (1).

Localization in the small intestine is rare; the incidence varies between 0.5 and 2.3%. It is usually identified at the level of the proximal jejunum (75%), with the ileum being identified only in 5% of cases (1). Jejunal diverticula are usually multiple and occur more frequently in men in the sixth or seventh decade (2, 3).

Diverticula form in the intestinal wall, in areas of low resistance, due to increased intraluminal pressure (4). They may have a genetic determinism that should be suspected, especially in the case of diffuse forms (5, 6). It is located more frequently in the jejunum than the ileum because the penetrating jejunal arteries have a larger diameter. Other favorable factors for the appearance of diverticles are dysmotility and abnormalities in the mesenteric plex (1, 4). They differ from Merkel diverticula, because they appear on the mesenteric margin (1).

Clinically, jejunal diverticulosis is usually asymptomatic (80%) of cases. When it is symptomatic, it is manifested by nonspecific abdominal pain, transit disorders (diarrhea/constipation), and flatulence (1, 7). Complications of jejunal diverticulosis occur in about 10% of cases, most commonly consisting of acute diverticulitis, mechanical obstruction, volvulus, perforation, peritonitis, and hemorrhage (1, 4). Perforation with peritonitis can be caused by an inflammatory diverticulum or a ruptured diverticular abscess, as was the case with our patient.

Because of its nonspecific symptoms and because it is rare, jejunal diverticulitis is often misinterpreted as appendicitis, peptic ulcer, cholecystitis, Crohn’s disease, or colonic diverticulitis (8). To avoid misdiagnosis, which inevitably leads to delayed treatment, clinicians should be aware of this entity.

Abdominal ultrasonography is used to establish the diagnosis at the first stage. This can sometimes indicate a thickened intestinal wall, irregular-looking formations related to the intestine, hypoechogenic having a hyperechogenic center – characteristic aspect for diverticula, and hyperechogenic tissue around these formations, indicating infiltration of surrounding fat or air bubbles (9).

Computed tomography (CT) is more sensitive in the diagnosis of acute diverticulitis and its complications compared to abdominal ultrasound and is therefore preferred (1, 10).

CT scans identify diverticular inflammation characterized by peridiverticular edema and thickening of the diverticular wall (8). The presence of pneumoperitoneum is not a definite sign of peritonitis, because the thin wall of the diverticulum can allow air to pass through (9).

CT diagnosis is difficult and remains uncertain in advanced local forms, in which diverticula can no longer be identified due to extensive local inflammation that causes fluid and gaseous infiltration. Highlighting other diverticula on the mesenteric edge of the loop of the small intestine helps clarify the diagnosis (9).

Selective mesenteric angiography or CT angiography may be used to locate active bleeding in cases of jejunal diverticular hemorrhage (11).

Treatment of diverticulitis can be conservative and medical (antibiotic therapy, according to table) (10).

The most common bacterial etiology of diverticulitis are:

1. Enterobacteriaceae: *Escherichia coli*, Klebsiella sp., aerobic high gram-negative bacillus.
2. Bacteroides species.
3. Enterococcus species: *Enterococcus faecalis* most common, *Enterococcus faecium.*
4. *Pseudomonas aeruginosa*: 3–15% (12) (see Table 1).

**Table 1:** Antibiotic treatment in case of acute diverticulitis (12).

Community infections	Severe/nosocomial infections
1. Ceftriaxon 2 g IV 24 h + Metronidazole 500 mg IV 8 h	1. Piperacilin–Tazobactam 4.5 g IV at 6 h (continuous infusion for 4 h)
2. Ciprofloxacin 400 mg IV on 12 h or Levofloxacin 750 mg IV on 24 h + Metronidazole 500 mg IV on 6 h or 1 g on 12 h	2. Cefepime 2 g IV on 12 h + Metronidazole 1 g IV on 12 h
3. Moxifloxacin 400 mg IV at 24 h	3. Meropenem 1–2 g IV at 8 h
4. Amoxicillin–clavulanate 1.2 g IV at eighth	4. Imipenem 500 mg/Cilastatin 500 mg IV at 6 h
	5. Doripenem 500 mg at 8 h (infusion for 1 h)

The average duration of antibiotic treatment is 5–10 days. The criteria for discontinuing treatment are clinical improvement, normalization of leukocytes, and resumption of intestinal transit (12).

In the case of localized limited perforation, conservative management treatment may be indicated in hemodynamically stable patients (4, 7), with the caveat that surgery should be performed if clinical improvement is not achieved within 48–72 h. In patients with peridiverticular abscess, antibiotic treatment and image-guided drainage (CT) may theoretically be sufficient, depending on the size of the collection and the possibility of a percutaneous approach (8).

For perforated jejunal diverticula, with peritonitis, or in the case of abuse or significant bleeding, literature data recommend emergency laparotomy, segmental intestinal resection, and primary anastomosis to avoid complications (13). Resection should be limited to the intestinal loop with complicated diverticulum (local abscess, peritonitis, or bleeding) to prevent short bowel syndrome (4, 11). An exception is pan-jejunoileal diverticulosis, for which conservative treatment may be preferred. This is because surgery can lead to severe malnutrition (3).

Risk factors for unfavorable evolution are old age, comorbidities, delay in diagnosis, and duration interval between perforation and surgery (9).

No consensus exists on the therapeutic strategy and management of jejunales diverticulitis (8).

To determine the best diagnostic method for complicated jejunal diverticulitis and the most appropriate treatment, we searched PubMed, MedNar, and Cochrane Library electronic databases for literature reviews on cases of jejunal diverticulitis published between 1.01.2014 and 31.12.2023. We considered the following terms in the studies’ title or abstract: “jejunal diverticulitis.” We excluded studies in languages other than English and French and excluded articles that did not cover several cases. The results are summarized in Table 2.

**Table 2:** Management of patients with acute diverticulitis according to specialized reviews from 2014–2023.

Nr. crt.	First -Named Author	Years	Average Age (years)	Sex(M:F)	Symptoms	Radiologic Investigation	Treatment	Conclusions
1	Zafouri (8)	2015–2017	67	19:8	Intermittent abdominal pain, fever, nausea, vomiting, altered bowel habits, recurrent bowel obstructions	CT-scan	Resection-19 Conservative – 8	Treatment of complicated acute jejunal diverticulitis is based on surgery, which consists of resection of the affected intestinal segment with primary anastomosis. This intervention prevents relapses.
2	Scheese (5)	2014–2022	66	15:4	Abdominal pain, fever, nausea	CT-scan	Resection-10 Conservative – 9	We can choose medical or surgical treatment for jejunal diverticulitis. Early initiation of treatment with intravenous antibiotics could prevent a risky surgical procedure in elderly patients, in whom this type of diverticulitis is most common.
3	Ng (13)	2015 −2018	71	3:5	Abdominal pain, nausea and vomiting, altered bowel habits, fever	CT-scan	Resection-5Conservative- 3	Computed tomography is the primary investigation for the accurate diagnosis of jejunal diverticulitis. An algorithm is needed to select suitable patients for conservative management.
4	Leber (9)	2005–2015	78	11 - severe form of diverticulitis	Acute abdominal pain	CT-scan	Resection-8 Conservative-3	Acute jejunoileal diverticulitis is a rare condition and usually localizes jejunal. A detailed CT evaluation can differentiate mild forms that do not require surgical treatment from severe ones that present as complicated jejunal diverticulitis.
5	Johnson (14)	1999–2012	72	18	Complications of jejunal diverticulitis: inflammation, bleeding, obstruction or perforation		Laparotomy and resection-14 Conservative - 4	Although conservative management can be successful in most patients with complicated jejunal diverticulitis, surgery is required.
6	Horesh (15)	2010–2015	72,1	8	Six patients had a sealed perforation; only one demonstrated a distant pneumoperitoneum.		Conservative-7 patients Resection −1	Jejunal diverticulitis can initially be treated conservatively, but surgical treatment should be considered in case of severe complications.
7	Kumar (16)	2018	64	2	complicated jejunal diverticulitis: abdominal pain, melena	CT scan −1 laparoscopic- 1	Resection −2	An accurate preoperative diagnosis of diverticulitis complications helps with better surgical planning.
8	De Simione (17)	2018		527	diverticular bleeding, intestinal obstruction, perforation		Resection - 244 Conservative −283	A multidisciplinary approach to the patient (involving a radiologist, surgeon, and gastroenterologist) is necessary to make an early diagnosis. In the case of complicated jejunal diverticulitis, surgical treatment is usually required.
9	López Marcano (18)	2002–2015	76	5:7	abdominal pain, gastrointestinal bleeding	CT scan	Resection of intestinal and anastomosis −12	Jejunal diverticulitis is rare. The first clinical manifestation is due to a diverticular complication. Abdominal CT is of choice in the diagnosis of jejuno-ileal diverticulitis. The treatment of choice is resection of the affected segment.
10	Mazahreh (19)		66	3: 4	gastrointestinal bleeding	angiography	segmental resection −6 argon plasma coagulation −1	Jejunal diverticulosis may be associated with angiodysplasia, which causes digestive bleeding. The treatment of choice is segmental resection.

As Table 2 shows, a CT scan is more sensitive in diagnosing acute diverticulitis, but angiography is used in cases of gastrointestinal bleeding. Sometimes, exploratory laparoscopy is needed for diagnosis.

Thus, surgical treatment remains the management of choice in patients with jejunal diverticulitis, possibly due to late diagnosis in the complication phase (8).

In our case, due to advanced local inflammation, the diagnosis could not be established correctly by CT, and the abscessed and perforated intestinal diverticulum was misinterpreted as an internal hernia with intestinal occlusion. It was necessary to perform a laparotomy for diagnostic purposes and surgical treatment. The postoperative evolution was favorable, although the patient had an increased surgical risk of presenting multiple comorbidities.

## 4 Conclusion

Diagnosing complicated jejunal diverticulosis is complex and sometimes not accurately established, even by high-resolution imaging, such as a CT scan, the more sensitive diagnostic technique. Diagnostic laparotomy is necessary in these situations. Surgical treatment should be considered in complicated jejunal diverticulitis before severe complications develop.

## Author contributions

SC: Writing – original draft, Writing – review & editing, Investigation, Methodology. MM: Conceptualization, Supervision, Writing – original draft, Writing – review & editing. MS: Investigation, Writing – original draft, Writing – review & editing. FM: Investigation, Methodology, Writing – original draft, Writing – review & editing. RC: Writing – original draft, Writing – review & editing.
